# Chronic recurrent multifocal osteomyelitis mimicking a malignant bone tumor: a case report

**DOI:** 10.11604/pamj.2022.42.150.19399

**Published:** 2022-06-24

**Authors:** Oumnia Bencharef, Tarik Salama, Elmouhtadi Aghoutane, Redouane Elfezzazi

**Affiliations:** 1 Faculty of Medicine and Pharmacy of Marrakech, Cadi Ayyad University, CHU Mohammed VI of Marrakech, Marrakech, Morocco

**Keywords:** Chronic recurrent multifocal osteomyelitis, children, malignant bone tumor, case report

## Abstract

Chronic recurrent multifocal osteomyelitis (CRMO) is an unusual form of non-microbial chronic osteomyelitis. It is an exclusion diagnosis that can only be considered after ruling out infectious osteomyelitis and bone tumors. We report the case of a 13-year-old girl who was admitted for a painful swelling of the left thigh. Biological examinations did not find an inflammatory syndrome. X-rays and Magnetic Resonance Imaging were very suggestive of malignant bone tumor. But the biopsy disclosed a nonspecific osteomyelitis. Considering the strong suspicion of a malignant origin, a second biopsy was performed and confirmed the diagnosis of osteomyelitis. During the hospital stay, the patient developed a second location in the left humerus. Thus, the diagnosis of CRMO was retained. The patient was treated by NSAIDs with good clinical and radiological outcomes. This case report reminds the diagnostic challenge of this pathology that can mimic malignant tumor.

## Introduction

Chronic recurrent multifocal osteomyelitis (CRMO) is an unusual form of non-microbial chronic osteomyelitis. It was initially described by Giedion *et al*. in 1972 as “subacute symmetrical and chronic osteomyelitis”. It affects mainly children and adolescents and especially females. It is often considered as the juvenile form of the SAPHO syndrome (acronym for synovitis, acne, palmoplantar pustulosis, hyperostosis and osteitis) [1-3]. Chronic recurrent multifocal osteomyelitis is an exclusion diagnosis that can only be considered after ruling out infectious osteomyelitis and bone tumors [4]. We report a challenging case of a 13-year-old girl presenting CRMO with clinical and radiological features of a malignant bone tumor, and discuss the epidemiology, diagnostic modalities and therapeutic options of this rare entity.

## Patient and observation

**Figure 1:**
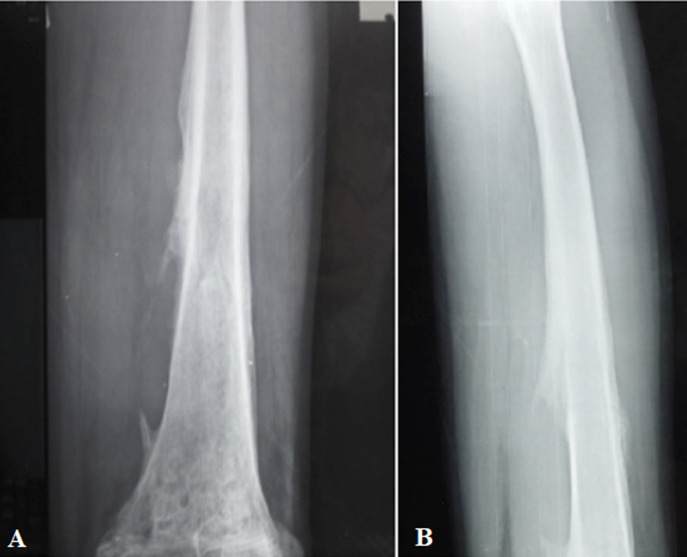
(A, B) anteroposterior and lateral X-ray of the left femur showing a moth-eaten osteolysis in the distal metaphysis extending to the epiphysis and the diaphysis, with periosteal reaction type Codman triangle and edema of the surrounding soft tissues

**Figure 2:**
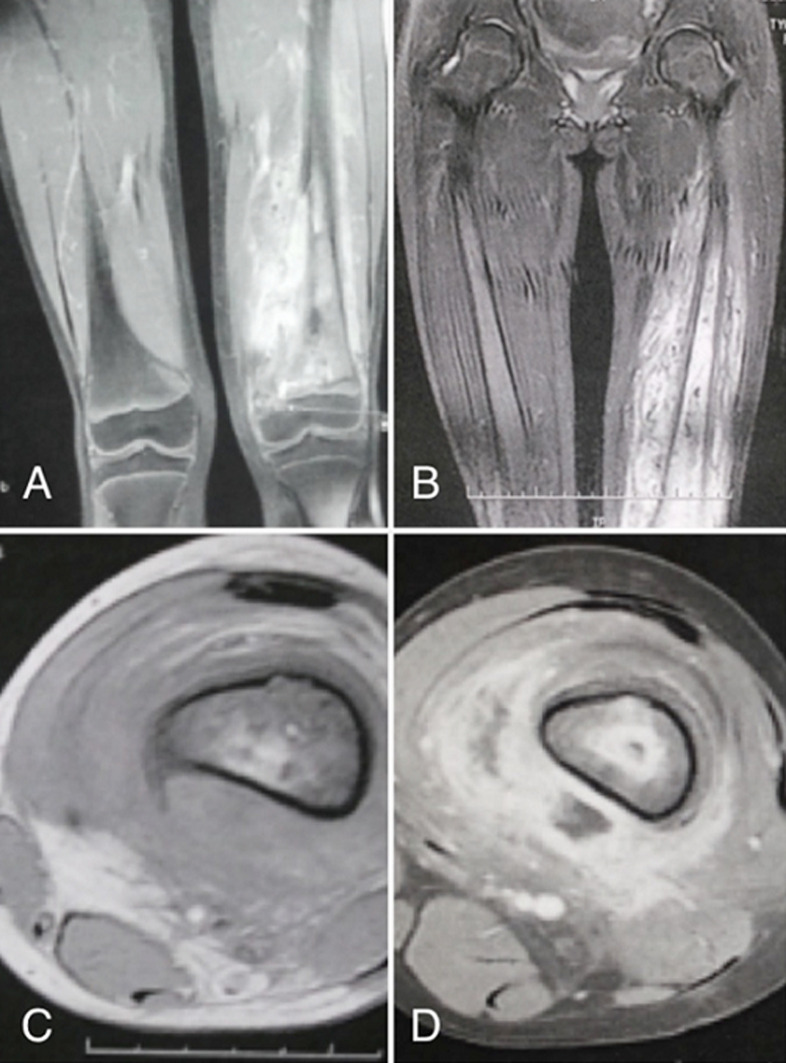
coronal T2 (A), STIR (B) and axial T1 weighted MRI without (C) and with contrast (D) showing metaphyseal-diaphyseal femoral process extending to the soft tissues and to the distal epyphysis

**Figure 3:**
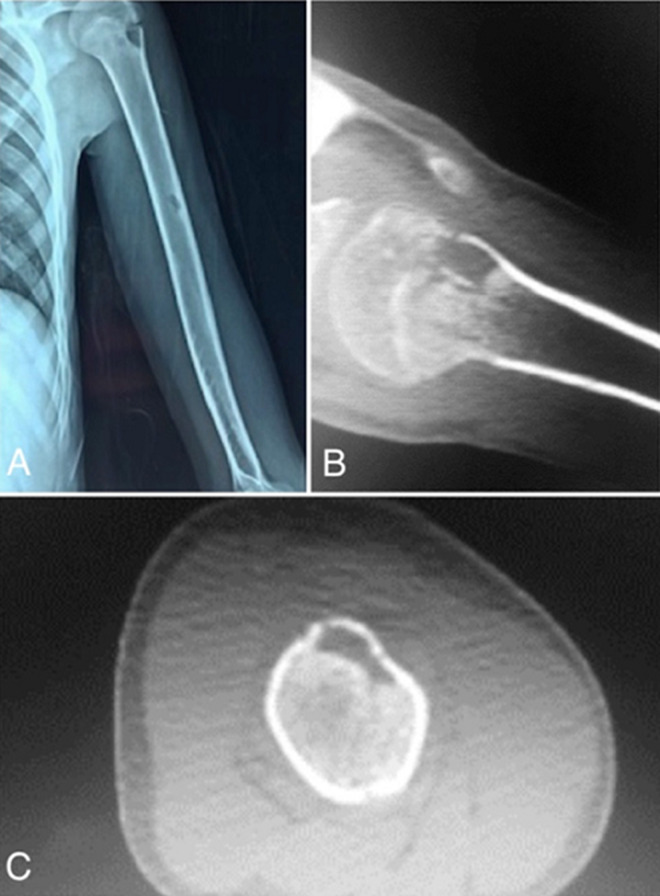
X-ray (A) and CT scan (B, C) of the left humerus showing small lytic diaphyseal and metaphyseal lesions, surrounded by marginal sclerosis

**Figure 4:**
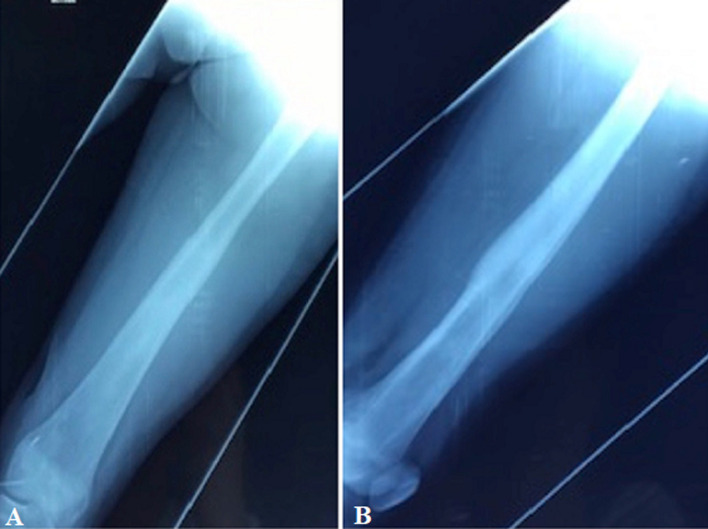
(A, B) X-ray control of the left femur after 2 years showing a complete resolution of the lesion

A 13-year-old girl was admitted in our department with painful swelling of the left thigh evolving for 3 months, without other symptoms. Physical examination found a hard and painful swelling of the distal part of the left thigh with knee stifness. The laboratory tests did not find an inflammatory syndrome. X-ray revealed a moth-eaten osteolysis in the distal metaphysis of the left femur, extending to the epiphysis and the diaphysis, with a Codman triangle type periosteal reaction and edema of the surrounding soft tissues (Figure 1). Based on this radiological appearance, a malignant bone tumor was first suspected. The patient underwent an MRI which was also suggestive of a malignant bone tumor (Figure 2). A surgical bone biopsy was performed at the lytic lesion. The pathological examination concluded to a nonspecific osteomyelitis, without malignancy signs. Considering the strong suspicion of a malignant origin, a second biopsy was performed and confirmed the diagnosis of osteomyelitis. Antibiotics were then started. During the hospital stay, the patient complained of a painfull stiffness of her left shoulder. The radiographs and the CT of the left humerus showed small lytic diaphyseal and metaphyseal lesions, surrounded by marginal sclerosis (Figure 3). Thus, the diagnosis of recurrent multifocal chronic osteomyelitis was retained based on the multifocality and the metaphyseal location of the lesions, and the absence of response to antibiotics. The patient was then treated by NSAIDs and showed a complete relief of here symptoms with good radiological outcome (Figure 4). She remained in complete remission during the two years of follow-up.

## Discussion

Chronic recurrent multifocal osteomyelitis is an aseptic bone inflammatory disease that affects the pediatric population, mainly girls (female to male ratio 2: 1). It usually occurs between the ages of 2 and 17 years old [2]. Its etiology is not yet well understood; a genetic and immunological origin have been suggested by some authors. The role of IL-1 in the development of sterile bone inflammation has been shown in recent clinical data [5]. The diagnosis of CRMO is based on clinical, biological and radiological arguments. It is a chronic illness with periodic exacerbations and remissions [6]. The evolution may extend over 2 to 20 years. The main clinical symptoms are pain and swelling, like experienced by our patient. Fever may occur in 50% of cases. Initially, the lesion involves most often one bone, but multifocal and symmetric lesions can be observed [3]. It mainly affects the metaphysis of long bones, especially the femur and tibia, followed by the clavicle then the spine [7]. Laboratory tests are not specific. An increased erythrocyte sedimentation rate (ESR) can be found, sometimes associated with leukocytosis [7,8]. Conventional radiography is the first required radiological examination; it may be normal at the early stage of the disease. The first radiological signs are changes in bone metaphysis close to the growth plates, while osteolytic lesions, sclerosis and hyperostosis usually appear in the late stages of the disease [3,7,8]. Our patient had, in addition to the osteolytic lesions, a Codman triangle like periosteal reaction, which was more suggestive of a malignant bone tumor. Magnetic resonance imaging (MRI) is a valuable supplement for the diagnosis and the evaluation of CRMO, and it is very sensitive in early stages of the disease. It usually demonstrates edema-like increased signal intensity in the medulla along with cortical thickening, periosteal reaction and edema in the surrounding soft tissue [3]. The MRI of our patient also evidenced signs of cortical bone breakout, which is also suggestive of a malignant bone tumor. Bone scintigraphy is helpful to assess the extent of lesions as some of them can be asymptomatic [9].

The role of the biopsy is still debated. In fact, the histological features are not specific, but it is very important to exclude all other causes of bone pain such as infectious osteomyelitis or, like in our case, a malignant bone tumor [10,11]. Some authors suggested that biopsy could be avoided if a child has classic radiographic findings of CRMO and specific comorbidities, such as Crohn's disease [11]. Clinical, biological and radiological criteria were also proposed to facilitate the diagnosis of CRMO and reduce the number of bone biopsies [5,10,11]. There are no guidelines regarding the management of CRMO, so the treatment remains Empiric. Most of authors agree that nonsteroidal anti-inflammatory drugs (NSAIDs) are the first-line treatment of CRMO. They reduce pain, induce remission and prevent bone damage [2,5,10]. They were very efficient for our patient since they allowed complete remission during the two years of follow-up. On-demand treatment seems as effective as long-term treatment. Their effectiveness is inconsistent; even if given continuously, NSAIDs may be insufficient. Corticosteroid therapy appears regularly effective for patients with CRMO who do not respond to NSAIDs, but its use is fraught with the usual complications in young children [4]. Methotrexate, been a well-known treatment in rheumatologic conditions, is a second-line treatment in CRMO, but further studies are needed to assess their effectiveness [7]. Bisphosphonates should be reserved for refractory cases or in patients with primary vertebral involvement and structural damage [12,13]. The evolution of CRMO and the factors that influence remission are still unclear. Some complications can occur such as epiphysiodysis, bone deformity, limb length discrepancy, fracture secondary to bone demineralization and kyphosis due to vertebral fracture [13,14].

## Conclusion

We reported the case of a girl with CRMO presenting with a confusing radiological appearance simulating a malignant bone tumor. The histological study and the multifocal character were essential to reach the diagnosis. This case report reminds the diagnosis difficulties of this still poorly known entity that can mimic malignant bone tumors.
